# Immunohistochemical Detection of Foot‐and‐Mouth Disease Virus in Carrier African Buffalo (*Syncerus caffer*)

**DOI:** 10.1002/vms3.70879

**Published:** 2026-03-13

**Authors:** Alischa Henning, Melvyn Quan, Angelika Loots, Lieza Odendaal

**Affiliations:** ^1^ Department of Paraclinical Sciences, Faculty of Veterinary Science University of Pretoria Pretoria South Africa; ^2^ Department of Veterinary Tropical Diseases, Faculty of Veterinary Science University of Pretoria Pretoria South Africa

**Keywords:** antigen, buffalo, carrier, foot‐and‐mouth disease virus

## Abstract

**Background:**

African buffalo are long‐term carriers of foot‐and‐mouth disease virus (FMDV), particularly the South African Territories serotypes. Detecting low viral loads in carrier animals remains challenging.

**Objectives:**

This study aims to detect FMDV antigen in formalin‐fixed tissues of carrier buffalo using immunohistochemistry (IHC) to identify potential sites of viral persistence.

**Methods:**

Polyclonal antibodies targeting structural FMDV proteins were used to detect FMDV in formalin‐fixed tissues from African buffalo (*n* = 15) culled in the Kruger National Park. The results were scored and compared with those from polymerase chain reaction (PCR).

**Results:**

Viral antigen was consistently associated with the vasculature of the coronary band and interdigital skin as well as in the lungs. It was also associated with the germinal centres of the palatine tonsils. Detection outside these areas was rare, indicating a distinct tissue tropism favouring the vasculature in carrier animals. All buffalo tested IHC positive, including some PCR‐negative cases, suggesting IHC may offer increased sensitivity when viral loads are low.

**Conclusions:**

The results revealed a novel association of viral antigen with vascular tissue and demonstrated the potential for superior sensitivity over PCR. Further research is needed to validate this assay, prove repeatability and clarify how carriers contribute to disease persistence and transmission.

## Graphical abstract

FMD viral antigen was consistently associated with the vasculature of the coronary band and interdigital skin as well as in the lungs. The IHC results revealed a novel association of viral antigen with vascular tissue and demonstrated the potential for superior sensitivity over PCR.

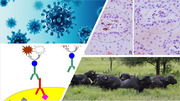

## 1 Introduction

Foot‐and‐mouth disease (FMD) virus (FMDV) belongs to the genus *Aphthovirus* within the *Picornaviridae* family with seven immunologically distinct serotypes (O, A, C, Asia‐1, Southern African Territories (SAT) ‐1, ‐2 and ‐3). It is a highly contagious, transboundary disease that affects both domestic and wild cloven‐hooved animals, and it is characterised by a high morbidity but low mortality rate (Stenfeldt and Arzt 2020). The virus replicates rapidly and spreads within infected animals and among in‐contact susceptible animals. Additionally, the inhalation of infected aerosols is the main transmission route, potentially causing long‐distance transmission events (Stenfeldt and Arzt 2020). Small, partially evaporated particles can remain infectious over time and can be dispersed over long distances (Brown et al. 2022).

Infected cattle clinically present with symptoms related to the presence of vesicular lesions in the oral cavity and/or on the feet. The disease has a range from mild to severe, with deaths only reported in young animals, that is, calves (Sahoo et al. 2022). The common clinical presentation includes pyrexia, anorexia, ptyalism, decreased habitus and lameness. If present, vesicular lesions are usually located on the muzzle, nostrils, lips, gums, tongue and palate as well as the coronary bands and interdigital spaces. The teats and udder may also be affected (Burrows et al. 1971).

Carriers are animals that have recovered from infection but continue to harbour virus in the oropharyngeal fluid for more than 28 days post infection and are not necessarily contagious (Van Bekkum et al. 1959; Sutmoller et al. 1968; Sutmoller 1971). The existence of the potential carrier state of many known FMD hosts complicates the prevention and control of the disease. African buffalo are the most important FMDV carriers of the SAT serotypes in Southern Africa and therefore implicated in disease maintenance (Salt 1993; Vosloo et al. 1996; Vosloo et al. 2002). Buffalo rarely develop vesicular lesions, and in cases of mild disease resolution occurs within 7–14 days (Young et al. 1972; Thomson 1997; Vosloo et al. 2007; Cortey et al. 2019).

Due to the highly contagious nature of the virus, regulations restrict the movement of fresh tissue, a critical measure of control in managing the disease. Consequently, diagnosis and research are confined to Biosafety Level 3 (BSL‐3) laboratories. However, formalin fixation inactivates the virus, allowing safe transport of infected samples for subsequent testing and/or research without the risk of viral spread. Moreover, techniques such as polymerase chain reaction (PCR), immunohistochemistry (IHC) and in situ hybridisation (ISH) can be performed on fixed tissue in lower biosafety settings (BSL‐1 and BSL‐2).

According to the World Organisation for Animal Health (WOAH), FMD diagnosis relies on detecting viral nucleic acid, viral antigen or live FMDV with reverse‐transcriptase PCR and virus isolation considered gold standards. In carrier animals, viral titers are typically low, and virus recovery is inconsistent (Grubman and Baxt 2004). Visualising viral antigen within cells or tissues offers deeper insights into disease pathogenesis, an advantage over PCR or viral isolation. To date, work performed in order to microscopically localise FMDV RNA in tissues has mainly been done via the classic fluorescent antibody technique using confocal microscopy and ISH (Arzt et al. 2009). In contrast, the immunohistochemical detection of FMD remains limited to a few studies over the past two decades (Arzt et al. 2009; Arzt et al. 2011; Patchimasiri and Rodtian 2016; Sahoo et al. 2022). Thus, there is a clear need for further research focused on antigen visualisation with improved sensitivity.

IHC presents additional benefits over chromogenic methods like ISH, including simpler protocols and the capacity to screen large numbers of tissues more cost‐effectively. Therefore, the aim of this study was to evaluate the potential of using IHC to detect FMDV antigen in carrier buffalo and to identify potential sites of viral persistence.

## 2 Materials and Methods

### 2.1 Diagnostic Samples

Tissues from African buffalo (*n* = 30) were obtained from the Kruger National Park (KNP), South Africa. From this, 15 represented the test samples and 15 the negative control samples. The test buffalo (*n* = 15) were culled as part of the park's population control. Their FMD status was determined by RT‐PCR at the Agricultural Research Council‐Onderstepoort Veterinary Research (ARC‐OVR) following established protocols (Callahan et al. 2002; Innis et al. 2012). Serum samples for RT‐PCR were collected at the time of slaughter and subsequent tissue sampling, while PCR analysis was performed at the ARC‐OVR. Serotyping was not conducted, as African buffalo in the KNP are often co‐infected with multiple serotypes (Bronsvoort et al. 2008; Maree et al. 2014; Di Nardo et al. 2015; Maree et al. 2016; Lazarus et al. 2018). Negative control tissues were obtained from disease‐free African buffalo (*n* = 15) from northern KwaZulu‐Natal, South Africa. The animals were held in quarantine in the KNP and repeatedly tested PCR‐negative for FMDV at 30 ‐ 60‐day intervals over three rounds, with the final test conducted at the time of slaughter. All animals included were subadult females and between 2 and 4 years of age. Additionally, tissues from adult, female, crossbreed cattle (*n* = 6) experimentally infected with FMDV were included. They were slaughtered approximately 10 days post‐infection, with viral presence confirmed with RT‐PCR. The bovine (*n* = 1) with the lowest cycle threshold (Ct) value (Ct = 29) was used as a positive diagnostic control. The Research Ethics Committee of the Faculty of Veterinary Science (FVS; University of Pretoria [UP]) granted the research protocol of the present study clearance with the following numbers: REC161‐21 and V036‐18.

Collected tissues were fixed in 10% buffered formalin and transported to the histopathology laboratory at the Section of Pathology, Department of Paraclinical Science, FVS, UP, South Africa. Samples collected from all animals included: ear tip (left or right), eyelid (left or right), lip (top or bottom), tongue, oropharyngeal tissue, retropharyngeal lymph nodes (medial and lateral), palatine tonsil, lung (left and right), coronary band (all four limbs) and interdigital skin (all four limbs). Formalin‐fixed tissues were processed into formalin‐fixed, paraffin‐embedded (FFPE) blocks within 7 ‐ 14 days post‐collection following Department of Agriculture (DA)‐accredited protocols. To preserve tissue for future analyses and minimise trimming, blocks were serially sectioned at 4 µm thickness. Sections were mounted on Superfrost slides, dried and stored for 7 days to 3 months prior to analysis.

### 2.2 Analytical Controls

The conditions for the assay were optimised using FMDV‐positive and negative baby hamster kidney‐21 cells from ARC‐OVR as previously described (Henning et al. 2025a). Harvested cells were formalin‐fixed, suspended in histogel and transferred to the histopathology laboratory at the FVS and embedded into paraffin blocks. In addition, the positive and negative control blocks were included in each IHC round performed on the positive control bovine, the test buffalo and the negative control buffalo.

### 2.3 Antibody Preparation

Polyclonal rabbit FMD antibodies (SAT‐1, ‐2 and ‐3), prepared at the ARC‐OVR, were used as the primary antibodies for FMDV immunolabelling. These antibodies target structural proteins and primarily recognise conformational epitopes on VP1, with contributions from VP2 and VP3.

Antibody specificity and titers were assessed using an in‐house enzyme‐linked immunosorbent assay (ELISA) against SAT‐1, ‐2 and ‐3 viruses. The ELISA employed SAT antigens previously isolated at the ARC‐OVR and is based on serotype‐specific blocking of heterologous FMD antigen by antibodies in the test serum. Briefly, ELISA plates were coated with rabbit anti‐FMD antibodies, then incubated with sera premixed with FMD antigen. If specific antibodies were present in the serum, they blocked the antigen, preventing its binding to the coated antibody. Absence of specific antibodies allowed antigen binding, producing a positive color signal indicative of a negative test result. Antibody titers were expressed as the 50% end‐point titers, with sera ≥ 1.6 log_10_ classified as seropositive (Hamblin et al. 1986; Cloete et al. 2008; Lazarus et al. 2018).

Positive and negative cell culture controls prepared as described previously by Henning et al. (2025a) were tested with antibodies against all three SAT serotypes (SAT‐1, ‐2 and ‐3) to evaluate cross‐reactivity. Once cross‐reactivity was established, the assay was applied to the positive control bovine, test buffalo and negative control buffalo samples. The anti‐structural protein antibodies demonstrated excellent efficacy in detecting FMDV across all tested SAT serotypes.

### 2.4 IHC

IHC was performed within the BSL‐1 facility of the Section of Pathology, Department of Paraclinical Studies, FVS, UP, South Africa. FMDV IHC was performed using a micro‐polymer kit (Mouse and Rabbit Specific HRP/DAB Detection Kit; Abcam 236466) with a NovaRed substrate (Vector SK‐4800) and hematoxylin counterstain.

The wax‐embedded tissue sections adhered to positively charged Superfrost glass slides. These were placed in a 40°C oven overnight. Thereafter, slides were placed in xylene for 10 min and then rehydrated in 100%, 96% and 70% ethanol for 3 min. Slides were triple rinsed in distilled water and placed in 3% hydrogen peroxide in methanol to quench endogenous peroxidase activity for 15 min.

For antigen retrieval, slides were microwaved in tris‐EDTA (TE) buffer (pH 9) at 96°C and thereafter allowed to cool on the bench at room temperature for 10 min. Slides were rinsed in distilled water three times followed by 0.1 molar (M) phosphate‐buffered saline (PBS), pH 7.6, containing 0.1% bovine serum albumin (BSA) buffer solution, rinsing for 10 min followed by incubation with the primary antibody at a dilution of 1/500 for 2 h.

Slides were rinsed three times in distilled water followed by 10 min in PBS‐BSA. The slides were further treated with Mouse and Rabbit Specific HRP/DAB Detection Kit (Abcam 236466). The mouse specifying reagent was incubated for 20 min and the anti‐rabbit HRP‐conjugate for 30 min. Slides were again rinsed three times in distilled water, repeated three times and then rinsed in PBS‐BSA buffer for 10 min. Using the Vector NovaRed kit, slides were checked under the microscope and then counterstained with Mayer's hematoxylin for 20 s. Last, the slides were rinsed under running tap water for 10 min, dehydrated through increasing ethanol concentrations, cleared in xylene, mounted in Entellan and fitted with a coverslip. In all cases, positive labelling was red‐brown and fine granular.

### 2.5 Interpretation

A strong cell‐associated signal was required for a positive IHC result without a similar signal in the negative control samples. A positive IHC reaction was characterised by red‐brown, often fine to coarse granules inside cells and tissues. Positive labelling was scored on a semi‐quantitative scale as negative (0/1), equivocal (±/1), which is a slight increase over the background, isolated (+/2), scattered positive (++/3) or widespread (+++/4). A light microscope (Olympus BH‐2) was used to determine the presence of IHC labelling, compared to the PCR results per case. The intensity of the immunoreactivity was not included within the scope of the work, considering that the IHC was performed manually. Sections were scanned with a Motic Easyscan Pro 6 Digital Slide Scanner. The digital images were evaluated using the Motic DSAssistant digital software.

## 3 Results

Twelve of the 15 test buffalo were PCR positive for FMDV, and three were negative. The IHC results mirrored the PCR results for 12 cases except for buffalo numbers 2, 4 and 6, which were also IHC positive (interdigital skin, coronary band, palatine tonsils and lungs) despite the negative PCR results (Table 1).

**TABLE 1:** Outcome of PCR vs. IHC in the test buffalo.

Buffalo number	PCR result	IHC result
Experimental cases		
1	+	+
2	—	+
3	+	+
4	—	+
5	+	+
6	—	+
7	+	+
8	+	+
9	+	+
10	+	+
11	+	+
12	+	+
13	+	+
14	+	+
15	+	+
**Total positive cases**	**12/15**	**15/15**

*Note*: The FMDV positive and negative control blocks were included in each IHC batch and consistently yielded IHC‐positive and IHC‐negative results, respectively (Figure 1).

**FIGURE 1:**
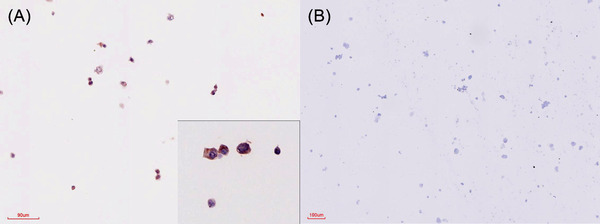
IHC results in FMDV‐positive and negative cell culture controls. (A) Red‐brown, intracytoplasmic, granular labelling in a positive cell culture section; the inset shows enlarged detail of the same section at the same magnification. (B) No staining in a cell culture section that was not infected with FMDV.

**FIGURE 2:**
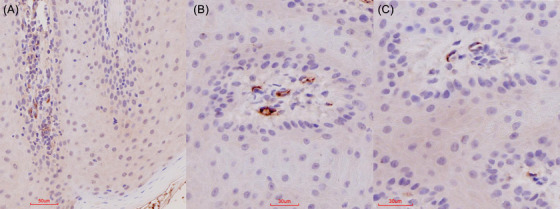
Vasculature‐associated IHC results in FMDV‐positive carrier buffalo and PCR‐negative buffalo no 2 and 6. (A–C) Red‐brown granular labelling associated with the vasculature in various locations of the interdigital skin and the coronary band in carrier buffalo and buffalo numbers 2 and 6.

**FIGURE 3:**
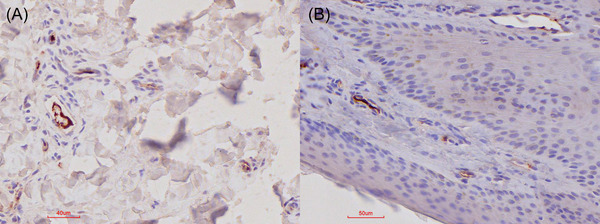
Vasculature‐associated IHC results in FMDV‐positive carrier buffalo. (A and B) Red‐brown granular labelling associated with the endothelium of smaller vasculature, that is, often arterioles in various locations of the interdigital skin and coronary band but often in the superficial dermis.

Immunohistochemical (IHC) positive labelling was present in PCR‐negative test buffaloes’ numbers 2, 4 and 6 as well as PCR‐positive buffaloes (Table 2). This was primarily limited to the interdigital skin and coronary band samples of all four feet (Table 2 and Figure 2). Positive labelling in the skin and lungs was associated with the endothelium (Figures 2 and 3), especially of the small blood vessels, that is, mainly arterioles.

**TABLE 2:** Outcome of IHC per location in the test buffalo group.

Buffalo number	IHC result	Tonsils	Lungs	Interdigital skin	Coronary band	Tissue mean
1	+	+ (2)	± (1)	++ (3)	+ (2)	**2**
2	+	± (1)	± (1)	+ (2)	± (1)	**1.25**
3	+	+ (2)	± (1)	+ (2)	± (1)	**1.5**
4	+	+ (2)	± (1)	++ (3)	+ (2)	**2**
5	+	+ (2)	± (1)	± (1)	+ (2)	**1.5**
6	+	± (1)	± (1)	± (1)	+ (2)	**1.25**
7	+	± (1)	± (1)	+ (2)	+ (2)	**1.5**
8	+	± (1)	± (1)	++ (3)	++ (3)	**2**
9	+	+ (2)	± (1)	++ (3)	+ (2)	**2**
10	+	+ (2)	± (1)	+ (2)	+ (2)	**1.75**
11	+	+ (2)	± (1)	± (1)	+ (2)	**1.5**
12	+	± (1)	± (1)	+ (2)	± (1)	**1.25**
13	+	± (1)	± (1)	++ (3)	± (1)	**1.5**
14	+	+ (2)	± (1)	± (1)	+ (2)	**1.5**
15	+	± (1)	± (1)	+ (2)	± (1)	**1.25**
**Tissue mean**		**1.5**	**1**	**2.07**	**1.73**	

*Note*: The IHC for FMDV were scored on a semi‐quantitative scale as negative (−/0), equivocal (±/1), which is a slight increase over the background, isolated (+/2), scattered positive (++/3) or widespread (+++/4). There was no IHC‐positive labelling in the pharyngeal tissue, retropharyngeal lymph nodes, ear, eyelid, lip and tongue in any of the test buffalo. PCR‐negative but IHC‐positive buffalo were represented by buffalo numbers 2, 4 and 6.

**FIGURE 4:**
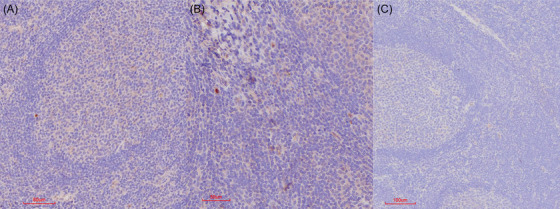
IHC results in tonsils in FMDV‐positive carrier buffalo. (A and B) Red‐brown granular, intracytoplasmic, cell‐associated labelling associated with the tonsillar germinal centres compared to (C) no IHC positive labelling in PCR‐negative buffalo.

**FIGURE 5:**
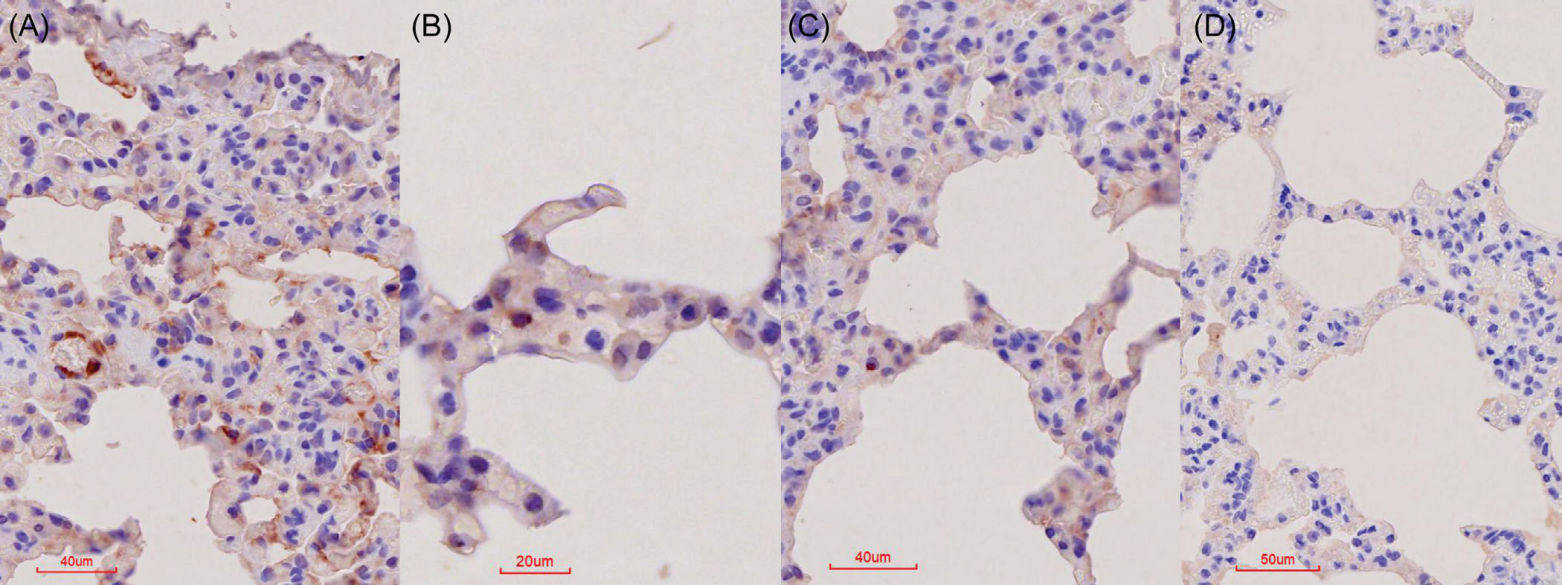
IHC results in lungs in FMDV‐positive carrier buffalo. (A–C) Red‐brown granular labelling in the lungs with (A) labelling associated with the pulmonary vasculature (B and C) Cell‐associated positive labelling in alveolar walls compared to (D) no IHC positive labelling in PCR‐negative buffalo.

In addition, positive labelling was associated with the tonsillar germinal centres and the alveolar walls of the lung (Figures 4 and 5). Positive labelling in the lymphoid tissue was cell‐associated, whereas the exact cell‐associated location within the alveolar walls in the lungs is speculative.

**FIGURE 6:**
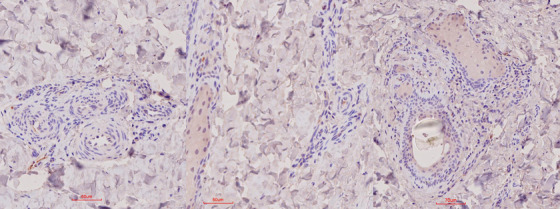
IHC results for the skin (interdigital skin and coronary band) in FMDV‐negative buffalo. Note the absence of specific labelling convincingly associated with the vasculature.

No labelling could, however, be observed in the remaining tissue, which included oropharyngeal tissue, retropharyngeal lymph nodes, lip, ear tip, eyelid and tongue. In the positive control bovine, IHC‐positive labeling was similar to that seen in the test buffalo but more extensive (Table 2). The negative control buffalo did not show any appreciable IHC‐positive labelling of any of the tissue samples included in the study (Figure 6).

## 4 Discussion

Immunohistochemical (IHC) detection of FMDV in animal tissues is a relatively novel diagnostic approach, explored in only a few studies (Zhang and Kitching 2001; Arzt et al. 2009; Pacheco et al. 2010; Sahoo et al. 2022). Arzt et al. (2009) identified the nasopharyngeal tissue as the primary infection site and the lungs as key during the viremic phase in experimentally infected cattle. Sahoo et al. (2022) extended antigen localisation to include less commonly studied organs, such as endocrine glands. Zhang and Kitching (2001) demonstrated viral persistence in the pharyngeal and soft palate epithelium up to 72 days post‐infection in cattle, comparing ISH and IHC.

African buffalo can be FMDV carriers for extended periods ranging from 5 to 20 years in closed herds (Arzt et al. 2011). Serological studies by Thomson and colleagues determined that 98% of buffalo in the KNP had been exposed to all three prevalent serotypes (SAT 1–3) as evidenced by an associated antibody response (Thomson et al. 1992; Thomson 1997). This supports our findings, where 15/15 buffalo tested positive for FMDV on IHC. Interestingly, while 15 buffalo were IHC positive, three were PCR negative. This suggests that IHC may offer improved sensitivity under conditions where viral titers are expected to be low. However, this will require further study to confirm. Additionally, given that clinical infections with FMDV in buffalo are rare with a subsequent paucity of infected cells/tissues, the positive and negative control blocks were included in each separate IHC batch, and the IHC results were effectively validated.

The exact role of carrier animals in FMD epidemiology remains controversial. This is primarily because the titers recovered from so‐called carrier animals decline over time and are often too low to be reliably detected or transmitted to naïve animals. (Donaldson and Kitching 1989; Juleff et al. 2008). Nonetheless, our findings indicate that IHC can identify viral persistence even in these low‐titer scenarios.

Sahoo et al. (2022) confirmed that the clinical signs, gross and microscopic lesions and antigen distribution were more prominent in cattle than buffalo, though their study compared clinically infected cattle with asymptomatic buffalo. In our study, a similar trend was observed. However, we compared a recovering, clinically infected bovine with carrier buffalo, which may not be directly comparable to each other or to the conditions in their study. Variations in viral strain, timing of sampling and host‐specific immune responses likely contribute to these differences in antigen distribution. FMDV antibodies target both structural and non‐structural proteins with capsid‐specific antibodies being serotype‐specific (Stave et al. 1986; Baxt et al. 1989; Monaghan et al. 2005; Yang et al. 2007). The VP1 gene is particularly valuable for diagnostic testing in South Africa due to its genetic variability among the FMDV serotypes and relative consistency within the SAT serotypes. (Maree et al. 2014; Lazarus et al. 2018). Our results successfully used VP1‐based epitopes to localise SAT antigens in carrier buffalo, revealing notable cross‐reactivity across all three SAT serotypes, supporting the use of a single serotype‐specific SAT antibody for broader SAT detection.

The virus employs various integrins, specifically α_V_β_1_, α_V_β_3_, α_V_β_6_ and α_V_β_8_ as in vitro cellular receptors via a highly conserved RGB acid amino located within the βG‐βH loop of VP1 (O'Donnell et al. 2009). While the connection between integrin expression and viral replication is recognised, the mechanisms remain incompletely understood. O'Donnell et al. (2009) reported that integrin α_V_β_6_ are expressed by epithelial cells, such as in the tongue and coronary band, that are important sites for FMDV replication. In contrast, α_V_β_3_ is associated with endothelial cells throughout various tissues but not in tongue keratinocytes, suggesting that α_V_β_6_ is central to viral tropism (Monaghan et al. 2005; O'Donnell et al. 2009). Additionally, Brown et al. (2022) reported that α_V_β_6_ is expressed in the respiratory airways of ruminants, aligning with the widely accepted theory that respiratory epithelium serves as an entry and replication site for FMDV. Integrin α_V_β_3_, due to its vascular association, may serve a secondary role by aiding viral dissemination (O'Donnell et al. 2009). Our work supports this hypothesis, particularly in the context of viral persistence in carrier animals and suggests that integrins may contribute to long‐term viral maintenance. However, the possibility exists that FMDV may use alternative membrane molecules as viral receptors and/or that the integrin expression does not solely dictate infection susceptibility and/or maintenance.

To our knowledge, this is the first report suggesting a strong association between viral persistence and blood vessel endothelium in carrier animals, especially of the interdigital skin and coronary band. Previous studies revealed viral persistence in the epithelial cells intimately associated with the underlying lymphoid tissue, especially within the oropharynx (Zhang and Kitching 2001; Alexandersen et al. 2002). It is proposed that follicular dendritic cells play a significant role in the long‐term immune response and persistence of FMDV, given their role in antigen trapping and subsequent presentations within lymphoid follicles. Follicular dendritic cells are in secondary lymphoid organs, trapping antigens and displaying them to B lymphocytes. They are also found within the germinal centres of lymphoid follicles in mucosa‐associated lymphoid tissue and lymph nodes. Maree et al. (2016) demonstrated higher viral persistence in the lymphoid tissue compared to the overlying epithelium in carrier buffalo. However, even in these tissues, viral presence was ultimately restricted to the pharyngeal tonsils and disappeared over time. Very little work has been performed on alternative sites of persistence, particularly outside of the head and neck lymphoid tissue. In the lungs, the dense vascular network may facilitate prolonged viral presence/persistence (Arzt et al. 2009; Arzt et al. 2010). Our results suggest a key role for the vascular system in the maintenance of infection, especially in carrier animals.

The difference between our findings and those of Henning et al. (2025a), who employed BaseScope to detect FMDV RNA in the same set of tissues in buffalo, may be attributed to several methodological and biological factors. Viral RNA was primarily present in the tonsils and lungs, compared to the current study, where IHC labelling was especially prominent in the interdigital skin and coronary band. (Henning et al. 2025b). This may be because IHC and ISH target different molecular components, where IHC detects viral proteins, whereas ISH targets specific nucleic acid sequences of DNA or RNA. Thus, it may reflect a distinct stage of infection or persistence. ISH is especially sensitive for detecting residual viral nucleic acids within epithelial or lymphoid tissues, whereas IHC may preferentially detect viral proteins persisting within the endothelial cells or associated macrophages (Maglennon and Doorbar 2012; Gheban‐Roșca et al. 2024). Variations in tissue fixation can alter tissue preservation and tissue morphology, while probe or antibody specificity influences sensitivity and the likelihood of cross‐reactivity. Additionally, the stage of infection at the time of sampling, affects viral replication and tissue distribution, potentially resulting in different detection outcomes between the two methods. Host factors such as immune modulation during the carrier state or differences in viral replication kinetics may also influence the relative distribution of antigen versus RNA signals across tissues. In conclusion, our work highlighted a strong relationship between the vascular system and FMDV persistence in carrier animals with strong IHC labelling in tissues like the interdigital skin and coronary band. Further investigation is necessary to confirm these findings and to fully elucidate the mechanism involved; a large sample size would also be beneficial to enable calculation and comparison of the sensitivity and specificity of IHC and ISH.

## Author Contributions

All authors contributed to the study conception and design. Alischa Henning performed material preparation, data collection and analysis. Alischa Henning wrote the first draft of the manuscript, and all authors commented on previous versions of the manuscript. All authors read and approved the final manuscript.

## Funding

AgriSETA supported this work with Grant Number PS22UP23.

## Ethics Statement

The Research Ethics Committee of the Faculty of Veterinary Science (University of Pretoria) granted the research protocol of the present study clearance with numbers: REC161‐21 and V036‐18. The directorate of the Department of Agriculture (DA) also granted permission for the study to be performed under Section 20 of the Animal Diseases Act, 1984 (Act No. 35 of 1984) with reference: 12/11/1/1/5 (2127 LH).

## Conflicts of Interest

The authors declare no conflicts of interest.

## Data Availability

The datasets generated and analysed during the current study are available from the corresponding author on reasonable request.
